# Unveiling the Remarkable Antioxidant Activity of Plant-Based Fish and Seafood Analogs through Electrochemical Sensor Analysis

**DOI:** 10.3390/bios13070751

**Published:** 2023-07-21

**Authors:** Gabriella Magarelli, Cínthia Caetano Bonatto, Gabriela Mendes da Rocha Vaz, Victoria Baggi Mendonça Lauria, Luciano Paulino Silva

**Affiliations:** 1 Embrapa Recursos Genéticos e Biotecnologia, Laboratório de Tecnologias para a Segurança Alimentar (LSA), Parque Estação Biológica, Final W5 Norte, Brasília 70770-917, DF, Brazil; gabriella.magarelli@embrapa.br; 2 Embrapa Recursos Genéticos e Biotecnologia, Laboratório de Nanobiotecnologia (LNANO), Parque Estação Biológica, Final W5 Norte, Brasília 70770-917, DF, Brazil; cinthiabonatto@gmail.com (C.C.B.); gabimendvaz@gmail.com (G.M.d.R.V.); victoriabaggi@gmail.com (V.B.M.L.)

**Keywords:** salmon analog, caviar analog, plant-based foods, voltammetry, glassy carbon electrode, antioxidant capacity, 3D printing, isoflavones, isolated soy protein, white beans

## Abstract

The global consumption of vegan foods is experiencing an expressive upward trend, underscoring the critical need for quality control measures based on nutritional and functional considerations. This study aimed to evaluate the functional quality of caviar and salmon analog food inks based on pulses combined with nano ingredients and produced in our laboratory (LNANO). The primary objective of this work was to determine the total antioxidant compounds contained in these samples using a voltammetric technique with a glassy carbon electrode. The samples underwent ethanolic extraction (70%) with 1 h of stirring. The voltammograms were acquired in a phosphate buffer electrolyte, pH 3.0 with Ag/AgCl (KCl 3 mol L^−1^) as the reference electrode and platinum wire as the auxiliary electrode. The voltammograms revealed prominent anodic current peaks at 0.76–0.78 V, which are attributed to isoflavones. Isoflavones, known secondary metabolites with substantial antioxidant potential commonly found in pulses, were identified. The total isoflavone concentrations obtained ranged from 31.5 to 64.3 mg Eq genistein 100 g^−1^. The results not only validated the efficacy of the electrochemical sensor for quantifying total antioxidant compounds in the samples but also demonstrated that the concentration of total isoflavones in caviar and salmon analogs fell within the expected limits.

## 1. Introduction

In recent years, the over-exploitation of seafood populations to meet global market demands resulted in detrimental environmental impacts, including biodiversity depletion, water eutrophication, coral reduction, and pathogen invasions, among others [1]. Furthermore, growing concerns about population health have driven the development of plant-based meat alternatives [2]. In recent years, the market for plant-based foods experienced expressive growth, such as in Brazil, where vegetable substitutes for meat and seafood witnessed a 30% increase in sales, reaching USD 107 million in 2021 compared to the previous year [3]. In response to this trend, new processing technologies, including spherification and 3D food printing, have been adopted to produce innovative plant-based food.

Spherification, a technique widely used in molecular gastronomy, enables the creation of products with high added value [4]. It involves the formation of edible spherical elements by extruding liquid through dripping. In this method, a highly viscous polymeric liquid (e.g., alginate and agar, among others) is extruded through a nozzle, forming droplets when applying an external or gravitational force and submerging into a bath solution [4]. Concurrently, more complex 3D printing techniques, which are primarily extrusion-based, are rapidly advancing. This process involves extruding a solution (food ink) with the desired characteristics (e.g., nutritional composition and sensory attributes, among others) layer by layer from a pre-designed digital model to form a 3D structure [5].

Despite the utilization of innovative processing technologies, developing fish and seafood analogs with nutritional profiles similar to animal-based counterparts remains challenging, primarily due to the limited nutritional value of vegetable proteins. Consequently, pulses emerged as intriguing sources for producing alternative meats, given their notable nutritional characteristics such as s protein content (~24%) and carbohydrate content (~63%) [6]. Pulses are also rich in essential amino acids including lysine, cysteine, and methionine [6]. Additionally, reports in the literature highlight the presence of phenolic compounds in pulses, such as phenolic acids, flavonoids, isoflavones, and tannins [6,7]. The antioxidant capacity of phenolic compounds, attributed to their reducing properties and chemical structure (benzene ring or cyclic structure with one or more hydroxyl substituents that interact with free radicals), contributes to their role in reducing the risk of chronic diseases, including coronary heart disease, diabetes, and cancer [7].

Extensive research has been conducted on phenolic compounds and their health benefits over the years [8,9]. Polyphenols are plant secondary metabolites commonly found in various fruits, vegetables, and plant-based foods. The development of analytical methods for their separation and characterization is of relevant interest due to their bioactive properties, health benefits, and potential use as natural antioxidants in the food, pharmaceutical, and cosmetic industries [8]. Spectrophotometry (UV-Vis), chromatography, and mass spectrometry are commonly employed analytical tools for polyphenolic analyses [10,11,12]. Spectrometric techniques, in particular, offer advantages such as high sensitivity, selectivity, speed, and the ability to identify and quantify multiple molecules simultaneously. Spectrophotometry is widely used to detect and quantify different classes of phenolics by utilizing compounds that react with antioxidants, leading to color changes. They are also valuable for evaluating the antioxidant capacity of matrices [13]. Electroanalytical techniques, including voltammetric methods, present an alternative approach for detecting and quantifying total phenolics or specific phenolic classes. They offer advantages over conventional methods, such as high sensitivity with low limits of detection and quantification, reduced solvent consumption, lower production of chemical residues, cost-effectiveness, portability, and the ability to assess antioxidant capacity [14,15,16].

Given the established health benefits associated with plant-based antioxidants and the growing consumption of pulse-based meat analogs, it is crucial to enhance the quality control of these foods by providing precise and comprehensive information about their antioxidant content. This research aims to propose a methodology based on differential pulse voltammetry and a glassy carbon electrode for determining the total phenolic compounds in fish (salmon) and seafood (caviar) analogs and evaluating the antioxidant capacity of these food products. Furthermore, this study seeks to contribute to the ongoing discussion on the use of electroanalytical techniques for the quality control of plant-based foods by analyzing additional functional compounds with antioxidant characteristics.

## 2. Materials and Methods

### 2.1. Materials

Analytical-reagent grade chemicals and ultrapure water were used to prepare solutions. Sodium alginate (ALG) was purchased from CRQ Química (Diadema, SP, Brazil), calcium chloride (CaCl_2_) was purchased from Dinâmica Contemporânea LTDA (Indaiatuba, SP, Brazil), white bean flour and isolated soy protein were purchased from Zona Cerealista (Vila Maria Alta, SP, Brazil), soy oil and annatto and saffron dyes were purchased from a local supermarket. Genistein (>99% purity) was purchased from L. C. Laboratories (Woburn, MO, USA). Phosphate buffers with a pH of 3.0–7.0 were prepared using dibasic sodium phosphate, monobasic potassium phosphate, and phosphoric acid (Sigma-Aldrich, Saint Louis, MO, USA) and used as supporting electrolytes. Ethanol and methanol (Sigma-Aldrich, Darmstadt, Hesse, Germany) were used for phenolic compound extraction in caviar and salmon analogs. Alumina powder < 10 μm (Sigma-Aldrich, USA), ethanol (Sigma-Aldrich, Saint Louis, MO, USA), ultrapure water, and nitric acid (Quimex, Uberaba, MG, Brazil) were used for glassy carbon electrode cleaning.

### 2.2. Preparation of Caviar Food Ink

**Figure 1:**
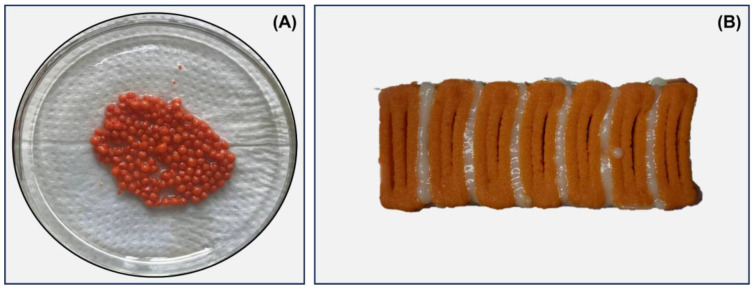
Caviar analog composed of white bean flour, soy protein isolate, and alginate produced via the spherification method (**A**). Salmon analog (fillet) composed of white bean flour and soy protein isolate produced via 3D printing (**B**).

Caviar analog (Figure 1A) was produced using the ionotropic gelation method using Ca^2+^ ions. ALG (1.25% *w*/*v*) was dissolved by stirring in filtered water for 30 min at room temperature. Simultaneously, an aqueous solution containing 22.5% *w*/*v* white bean flour and an oil solution composed of 45% isolated soy protein and annatto dye were produced. The dissolved substances were added to the alginate solution and then stirred for 10 min.

### 2.3. Preparation of Salmon Analog Food Inks

Two food inks (white and orange) were formulated to produce the salmon analog (Figure 1B). For the development of the orange ink, isolated soy protein (1.92 g) was solubilized for 10 min at room temperature in a nanoemulsion containing natural annatto and saffron dyes (solution 1). Then, white bean pulse flour (1.92 g) was solubilized in water following the same process and added to solution 1, remaining under stirring for 10 min. To develop white food ink, vegetable oil and water were used to solubilize the isolated soy protein (0.96 g) and white bean pulse (0.96 g), respectively, following the same methodology described above. Then, the solutions were mixed and stirred for 10 min. Finally, the food inks were added to 5 mL Luer Lock syringes for the 3D printing process.

### 2.4. Digital Manufacturing: Spherification and 3D Printing

For the development of caviar analog, the food ink was added to syringes with a 3 mL Luer slip nozzle and a hypodermic needle with a caliber of 21 G. The syringe was positioned on an automatic injector at a speed of 200 µL/min (water equivalent) and a height of 8 cm. Below the needle, a beaker containing 20 mL of a 1.5% *w*/*v* CaCl_2_ solution was placed to collect the ink, which formed structures that were left for 5 min, and then washed in distilled water.

For the salmon analog, a computer-aided design (CAD) model was developed in order to contemplate the use of two different food inks. The CAD model considered the two most distinct visual components of the salmon: a white-streaked part and a mostly orange part. The modeling was completed using the Tinkercad platform, and the gcode was generated from Slic3r. The bioprinter control was performed using Pronterface. For the 3D food printing process, 14 G needles without bevels were attached to 5 mL Luer Lock syringes, which were previously filled with the appropriate food ink. These syringes were then placed one after the other in the STARTER bioprinter (3D Biotechnology Solutions–3DBS).

### 2.5. Samples Extraction and Voltammetric Analysis

Phenolic compounds were extracted in quintuplicate from 0.1 g sample of fresh caviar and salmon analogs using Kline orbital stirring plate (1 h, room temperature) in 2.0 mL of 50% aqueous ethanol. The resulting extract was decanted and the supernatant was transferred to 2.0 mL microtubes and centrifuged for 10 min at 12,100× *g*. To optimize the experimental conditions and achieve the highest sensitivity for the differential pulse voltammetry (DPV) method, the influence of the solvent used for phenolics extraction (methanol and ethanol) and the solvent concentration (25, 50, 70%) on the total phenolics oxidation peak currents (Ipa) were studied.

The voltammetric measurements were conducted on a PGSTAT 128 N Autolab (Metrohm, Switzerland) with electrochemical software (NOVA 2.16, Metrohm, Switzerland). Voltammetric measurements were carried out in an electrochemical cell composed of a glassy carbon electrode (GC) (Φ = 2.0 mm) as the working electrode, Ag/AgCl (3 mol L^−1^ KCl) electrode as the reference electrode, and a platinum wire as the auxiliary electrode. Before each measurement and between sample measurements, the surface of the glassy carbon electrode was cleaned and electrochemically activated following this procedure: the electrode surface was manually polished with alumina suspension on a polishing cloth for 2 min and rinsed with distilled water; the electrode was immersed in ethanol for 5 min and in HNO_3_ 1:1 *v/v* for 1 min, then rinsed with ultrapure water; the electrode was immersed in phosphate buffer (0.2 mol L^−1^, pH 7.0) and subjected to ten potential cycling with cyclic voltammetry in the potential range between −1.5 and 1.5 V at a scan rate of 50 mV s^−1^.

The voltammetric profile of the samples was obtained in two different pH values by adding 500 and 800 µL of the extract of salmon and caviar analogs, respectively, to the electrochemical cell containing 10 mL of 0.2 mol L^−1^ phosphate buffer, pH 3.0 and 6.0. The extracts were analyzed via DPV with potentials ranging from 0 to 1.0 V at a pulse amplitude of 50 mV, step height of 5 mV, modulation time of 0.05 s, interval time of 0.1 s, and sweep rate of 50 mVs^−1^. The concentrations of total phenolic compounds in caviar and salmon analog samples were obtained from the standard additions of 5 µL of genistein 1.0 × 10^−3^ mol L^−1^ to the samples and expressed as mg of genistein equivalents per 100 g of samples (mg GEN 100 g^−1^). Stock solutions of genistein with concentrations of 1.0 × 10^−3^ mol L^−1^ were prepared using a mixture of water and ethanol (1:2) and stored in dark vials under freezing. Calibration curves of genistein were obtained by using DPV with six successive additions of 5 μL of 1.0 × 10^−3^ mol L^−1^ genistein to the electrochemical cell containing 10 mL of 0.2 mol L^−1^ phosphate buffer pH 3.0. The voltammetric measurements for each concentration level were performed in triplicate.

The intra-day precision (repeatability) of the DPV method was evaluated by measuring the relative standard deviation (RSD) of seven independent measurements of genistein oxidation current peaks at fixed concentrations of 0.5, 1.5, and 3.0 µmol L^−1^. The inter-day precision (intermediate precision) of the DPV method was evaluated by measuring the (RSD) value of the genistein oxidation current peaks at fixed concentrations of 0.5, 1.5, and 3.0 µmol L^−1^ on three different days. Linearity, the limit of detection (LOD), and the limit of quantitation (LOQ) for the DPV method were obtained from the genistein calibration curve [17]. The LOD was calculated using the expression LOD = 0 + t_(n−1.1−α)_ × s, where t is the student distribution for *n* = 10 independent sample blanks; α is the 95% confidence level, and s is the standard deviation of the sample blank values fortified at the lowest acceptable concentration measured for genistein. The LOQ was calculated from the lower end of the working range [17]. The trueness of the DPV method was assessed by recovery assays in which known amounts of genistein (0.5, 1.5, and 3.0 µmol L^−1^) were added to the caviar and salmon analog extracts.

### 2.6. Antioxidant Capacity Evaluation Using the Voltammetric Method

The antioxidant capacity evaluation of the samples was carried out using the electrochemical index (EI) value, calculated according to Lino et al. (2014) [18]. The EI was proposed considering the direct information that peak potentials (Eap) can provide on the sample reduction potential, and the peak current (Iap) can provide information about the concentration of antioxidants in the analytical sample. The quotient between Iap and Eap resulted in the EI value. The voltammetric parameters Iap and Eap were obtained from differential pulse voltammograms of 1 mL of caviar and salmon analog extracts in 10 mL of 0.2 mol L^−1^ phosphate buffer, pH 3.0, using the following electrochemical parameters: initial potential (Ei) = 0.0 V, final potential (Ef) = 1.0 V, pulse amplitude = 50 mV, scan rate = 50 mV s^−1^, working electrode: glassy carbon, reference electrode: Ag/AgCl (KCl 3 mol L^−1^), auxiliary electrode: platinum wire.

### 2.7. Statistical Analysis

The data from the optimization and validation experiments of the voltammetric method were summarized using Microsoft Office Excel (version 365). The voltammograms were constructed using the NOVA 2.16 software, suitable for operating the voltammetric analyzer. Specific statistical tests, such as the Grubbs test (used to remove outliers), and the Crochan test (used to test linearity), were performed following the bibliographic references indicated in the INMETRO document, 2020 [18]. The calculations of the mean, standard deviation (SD), and relative standard deviation (RSD) of the data sets were performed using Microsoft Office Excel software (version 365). Linear regression and the linear curve equation of the analytical curves were also obtained using Microsoft Office Excel software (version 365).

## 3. Results

### 3.1. Influence of Solvent on Sample Extraction

**Figure 2:**
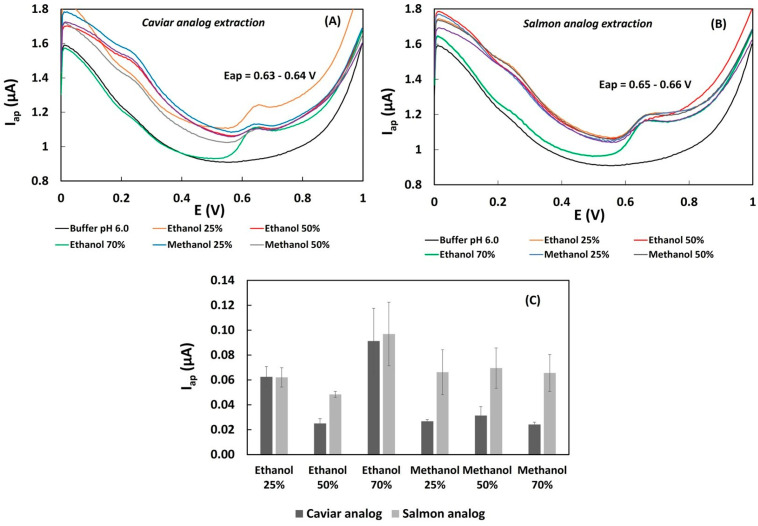
Influence of the hydro-organic solvent and its concentration on the extraction of phenolic compounds from samples. (**A**) Differential pulse voltammograms obtained from caviar analog extracts (obtained via extraction with 25–70% ethanol/methanol). (**B**) Differential pulse voltammograms obtained from salmon analog extracts (obtained via extraction with 25–70% ethanol/methanol). (**C**) Bar graph displaying the intensities of oxidation (anodic) peak currents–Iap (µA) samples extracted with ethanol and methanol at different concentrations.

**Figure 3:**
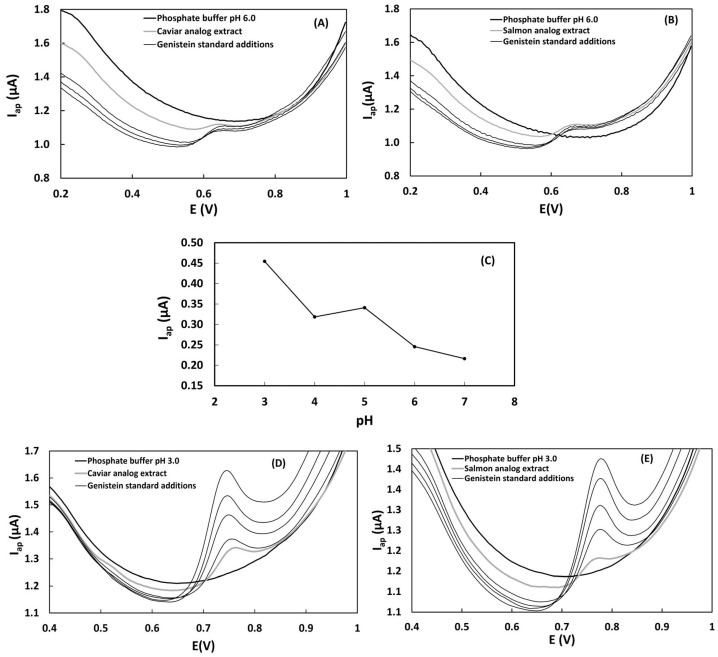
Influence of pH on the quantification of isoflavones. (**A**) Differential pulse voltammograms of caviar analog extract (70% ethanol) with standard additions of genistein 1.0 × 10^−3^ mol L^−1^ in pH 6.0. (**B**) Differential pulse voltammograms of salmon analog extract (70% ethanol) with standard additions of genistein 1.0 × 10^−3^ mol L^−1^ in pH 6.0. (**C**) Oxidation (anodic) peak current (Iap/µA) intensities of genistein standard 1.0 × 10^−3^ mol L^−1^ with different pHs. (**D**) Differential pulse voltammograms of caviar analog extract (70% ethanol) with standard additions of genistein 1.0 × 10^−3^ mol L^−1^ in pH 3.0. (**E**) Differential pulse voltammograms of salmon analog extract (70% ethanol) with standard additions of genistein 1.0 × 10^−3^ mol L^−1^ in pH 3.0.

In order to identify the optimal solvent for extracting phenolic compounds from caviar and salmon analogs samples with soy protein and white bean pulse constitution, aqueous methanol and ethanol were used for sample extraction at different concentrations (25%, 50%, and 70%). The selection of the suitable extraction solvent was based on the voltammetric profiles and anodic currents of the samples. Favorable results were characterized by well-formed voltammetric curves without overlapped peaks and higher anodic currents. The DPV profiles of the samples extracted with ethanol and methanol exhibited oxidation current peaks at potentials around 0.2 V and 0.6 V in phosphate buffer pH 6.0 (Figure 2A,B), indicating a similar polyphenolic composition). Figure 3C demonstrates that ethanol and methanol at various concentrations significantly influenced the extraction of total phenolic content in 0.1 g of fresh caviar and salmon analog samples. The highest intensity of the anodic current peak (Iap) at 0.6 V was observed in samples extracted with 2 mL of 70% ethanol (Figure 2C). Under these conditions, the extraction method showed a precision evaluated for 0.1 g of the salmon analog sample over three days, with a RSD of 25% (Figure 2C). Previous research on the voltammetric determination of isoflavones in soybean samples also reported promising results in using 70% ethanol [19].

### 3.2. Differential Pulse Voltammetry Measurements of Samples with Isoflavone Standard Additions at Different pH Values

The differential pulse voltammograms obtained for caviar and salmon analog samples with standard additions of genistein in 10 mL of 0.2 mol L^−1^ phosphate buffer; pH 3.0 and 6.0 are presented in Figure 3A,B,D,E. Under the established electrochemical conditions using the glassy carbon electrode, a well-defined anodic current peak was measured at 0.64–0.65 V in phosphate buffer at pH 6.0 (Figure 3A,B). In the voltammograms obtained in phosphate buffer pH 3.0, the anodic current peak was observed at potentials of 0.76–0.78 V (Figure 3D,E). This voltammetric profile can be attributed to the oxidation of total isoflavones, a class of polyphenolic compounds commonly found in soy-based foods [20]. Previous studies have reported similar voltammetric profiles with anodic current peaks at potentials ranging from 0.5 to 0.6 V for isoflavones such as genistein, glycitein, daidzein, daidzin, genistin, and glycitin [14,19]. This electrochemical behavior with the oxidation of a specific hydroxyl group located in the benzene B-ring of the isoflavones at the glassy carbon electrode surface was discussed in our previous work [14,19]. No voltammetric signal was observed in the analysis of the samples of annatto and saffron dyes used in the food ink of caviar and salmon analogs under the adopted experimental conditions. This observation suggests that the dye molecules may not be electroactive or detectable by the glassy carbon sensor. Additionally, the non-solubility of the dyes in 70% ethanol could be a contributing factor.

Genistein was chosen as the standard for determining total isoflavones due to its good performance as a quantitation standard for total isoflavones in soy-based food. To obtain more accurate results for the quantification of total phenolic compounds through the construction of a standard addition curve, it is important that the current increments during the addition of the standard are increasing and sensitive, resulting in a linear standard addition curve. The analysis conditions that resulted in more linear curves were obtained in phosphate buffer at pH 3.0. These results support the influence of pH on the determination of genistein. Differential pulse voltammograms of genistein (9.9 µmol L^−1^) showed more intense oxidation currents at pH = 3.0 (Figure 3C). Under these conditions, genistein proved to be a more suitable standard for the quantification of total isoflavones, as evidenced by the well-defined peaks of oxidation currents (Eap = 0.64–0.65 V) that perfectly overlapped the signals of the sample without any interference signals such as shoulders and double peaks (Figure 3D,E).

### 3.3. Quantification of Total Phenolic Compounds in Caviar and Salmon Analogs—Validation Parameters

**Figure 4:**
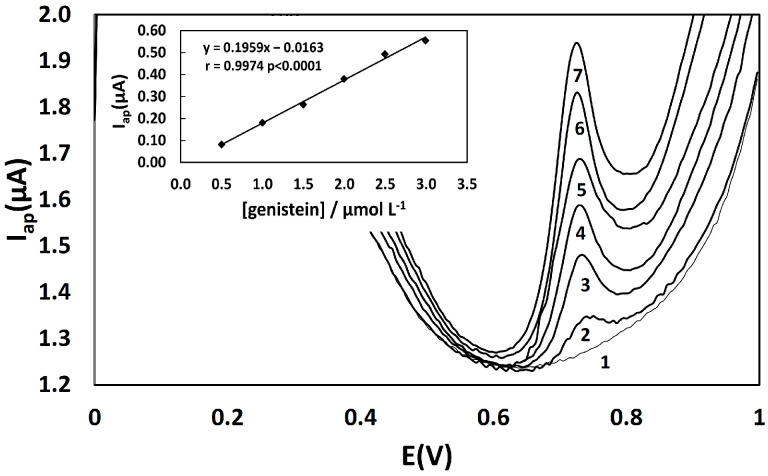
Differential pulse voltammograms for standard additions of genistein 1 × 10^−3^ mol L^−1^ in 0.2 mol L^−1^ phosphate buffer at pH 3.0. 1: Phosphate buffer at pH 3; 2–7: 0.5 µmol L^−1^, 1.0 µmol L^−1^, 1.5 µmol L^−1^, 2.0 µmol L^−1^, 2.5 µmol L^−1^, 3.0 µmol L^−1^ of genstein. Insert—the calibration curve (correlation coefficient (r) = 0.9974; probability (*p*) < 0.0001).

Table 1 summarizes the results of linearity, LOD, LOQ, and precision. The analytical curve for the successive addition of a genistein solution, prepared at 1.0 × 10^−3^ mol L^−1^ in 0.2 mol L^−1^ phosphate buffer at pH 3.0, was obtained using differential pulse voltammetry with a glassy carbon electrode. Differential pulse voltammograms exhibited an increase in the anodic current signal at 0.76–0.78 V with the addition of genistein (Figure 4). A linear curve of anodic current Iap (µA) as a function of genistein concentration (μmol L^−1^) was obtained in the range from 0.5 to 3.0 μmol L^−1^ (Insert of Figure 4), with a limit of detection of 0.1 µmol L^−1^ and a limit of quantitation of 0.5 µmol L^−1^. The linearity of the method within the working range was confirmed by a correlation coefficient (r) greater than 0.99 and the homoscedasticity of the analytical curve, as demonstrated by the calculated Cochran value (α = 95% confidence level) being lower than the theoretical value.

The intra-day precision (repeatability) of the DPV method exhibited an oxidation current with a RSD ranging from 4.8 to 5.6% based on seven independent measurements at three concentration levels of genistein. The inter-day precision (intermediate precision) of the DPV method showed an oxidation current with RSD of 7.4 to 9.6%, considering one independent measurement at three levels of genistein concentration in three different days. These results indicate that the method provides satisfactory intra-day and inter-day precisions in the voltammetric determination of total phenolics using genistein as an equivalent standard compound.

### 3.4. Concentrations of Total Phenolic Compounds in Caviar and Salmon Analogs and the Electrochemical Index (EI)

The concentration of total isoflavone compounds in caviar and salmon analogs, expressed as mg of genistein equivalents per 100 g of samples (mg GEN 100 g^−1^), was determined. The samples were prepared using extrusion and 3D food printing methods. The mean values and standard deviations are presented in Table 2. Standard addition curves were constructed by plotting the oxidation current peak against the added concentrations of genistein (0.5, 1.5, and 3.0 µmol L^−1^) in the caviar and salmon analogs. These curves exhibited good sensitivity (slope = 0.098–0.12) and a linear correlation (r > 0.99). The recovery range of added genistein in the caviar samples was 72–112%, while for the salmon analog, it ranged from 75 to 117%.

**Figure 5:**
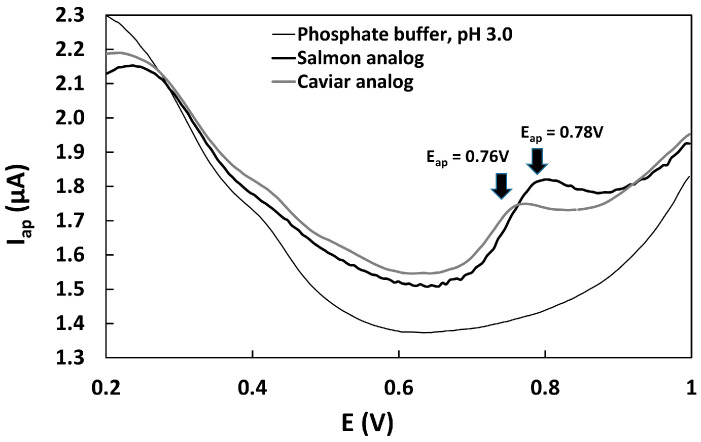
Differential pulse voltammograms of caviar analog and salmon analog extracts (in 70% ethanol) in phosphate buffer at pH 3.0.

The EI was calculated based on the voltammograms obtained from 1 mL of sample extracts (Figure 5). The averages of the anodic current peaks and anodic peak potentials (*n* = 3) were used in the calculation of the EI, which are listed in Table 2.

## 4. Discussion

The quality control of plant-based foods, such as fish and seafood analogs, primarily focuses on nutritional analysis, including protein, carbohydrate, and lipid content, which should be similar to their animal-based counterparts. However, it is equally important to consider the concentration of functional compounds in these foods. In our study, we expected to find isoflavones in caviar and salmon analogs based on the extensive literature highlighting the functional and health benefits of isoflavones in legumes such as soybeans and various types of beans [21,22,23,24,25,26]. Interestingly, this antioxidant class of substances is still underexplored as a quality control parameter in commercial plant-based foods.

Isoflavones belong to the flavonoid family and are an important subclass of phytoestrogens commonly found in legumes such as soybeans, beans, chickpeas, and sunflower seeds. They possess antioxidant properties and have the potential to benefit human health by reducing the risk of cancer, preventing obesity and diabetes, mitigating cardiovascular diseases, addressing osteoporosis, and alleviating menopausal symptoms [27]. In our study, isoflavones in the caviar beads and in the salmon fillet analog primarily originated from isolated soy protein in the food inks. The concentration of isolated soy protein in the salmon analog ink was higher than that in the caviar analog ink, contributing to its higher isoflavone content. However, it is worth noting that the concentration of isoflavones in soybean seeds is generally higher compared to soy protein isolates. Silva et al. reported total isoflavone content ranging from 165 to 336 mg/100 g in soybeans with different varieties from Brazil [28]. Rizzo and Baroni also reported isoflavone concentrations ranging from 99.82 mg 100 g^−1^ in soybeans from Brazil [21]. The variation in isoflavone content is attributed to different climatic conditions and cultivation practices.

During the processing of soy protein isolate, approximately 52% of the isoflavones are lost [23]. Liu et al. found isoflavones in samples of isolated soy proteins with a concentration of 62.5 mg 100 g^−1^ (acid leach) [23]. In their analyses using liquid chromatography, the acid leach processing to soy protein isolates improved the extraction of β-glycosides and aglycones isoflavones. However, even after processing, the concentration of isoflavones found in our study for caviar analog (31.5 ± 1.8 mg Genisteína 100 g^−1^) and salmon analog (64.3 ± 2.0 mg Genisteína 100 g^−1^) is higher compared to other reported soy foods. For example, soy burgers contain 0.1–26 mg 100 g^−1^ of total aglycone equivalents, soy cheeses 2.3–33 mg 100 g^−1^, and soy milk formulas can have up to 31 mg 100 g^−1^ [21].

Regarding the potential adverse effects of soy consumption, such as the disruption of the sex hormone network, infertility, and allergies, studies have yielded conflicting results with limited clinically confirmed investigations [29]. In 2015, the European Food Safety Authority (EFSA) panel concluded that the intake of 35–150 mg per day of isoflavones from supplements or foods does not have adverse effects. As a precautionary measure, it is recommended not to introduce soy foods beyond 6 months of age to prevent allergies [21].

In common beans, phenolic acids, flavonoids, and proanthocyanidins are the primary polyphenols, with pigmented seed coats containing higher polyphenol levels. However, white beans, which are present in the caviar and salmon analog samples, have a deficient presence of isoflavones. Ombra et al. reported total flavonoid content of approximately 5–6 mg 100 g^−1^ in white bean varieties [30]. Gharachorloo et al. found a concentration of total phenolics of 10.1 mg 100 g^−1^ in white bean seeds [26].

Our results from the DPV demonstrate that a bare glassy carbon sensor can effectively determine polyphenols with good performance. The LOD, linearity, precision, and recovery features of the method confirm its usability. However, it is important to note that the strong adsorption of phenolic compounds on the electrode surface required a longer cleaning process before each analysis, increasing the time required for electrochemical analysis. It is worth mentioning that voltammetric methods have limitations in selectively detecting phenolic compounds. In the voltammograms of the samples, sensitive oxidation current peaks were observed at the same potential. Hence, for quantitative determination, we adopted genistein, resulting in sensitive current peaks free from interferences. Drawing from our previous experience in determining isoflavones in soybeans, we were able to streamline the optimization process, considering parameters such as sweep speed, amplitude, electrolyte, and standards for quantification. All these optimized parameters were employed in the electrochemical analysis of isoflavone determination in caviar and salmon analogs. The evaluation of the sample’s antioxidant capacity using the EI revealed that the salmon analog exhibited better antioxidant capacity, primarily due to its slightly higher polyphenolic content, at the anodic peak potentials (0.76 V for caviar analog and 0.78 V for salmon analog). The use of EI is suitable for comparing the antioxidant capacity of a group of samples in the same study and analyzed using the same electroanalytical method. As EI depends on the electrochemical and experimental parameters, it cannot be directly compared in different studies. Additionally, good correlations have been observed between the antioxidant capacity values determined via EI using voltammetry and EC50 determined via spectrophotometry [18].

Looking ahead, future perspectives involve the utilization of lab-made portable sensors modified with nanostructures, such as green synthesized monometallic or bimetallic nanoparticles. These advancements will enhance the application of electrochemical sensors and address the issue of molecule adsorption from the extracts.

Furthermore, researchers are exploring various legume proteins derived from sources like fava beans, lentils, chickpeas, and peas as alternatives to soy protein. These studies aim to improve the nutritional value and provide advanced fish and seafood analogs.

## 5. Conclusions

The growing recognition of the health benefits associated with antioxidants from plants and the increasing consumption of plant-based meat analogs underscores the need to enhance the functional quality control of these foods. In this study, we successfully analyzed the antioxidant content of caviar and salmon analogs using a voltammetric technique with a bare glassy carbon electrode. The method exhibited high sensitivity, satisfactory precision, and low limits of detection and quantification, making it suitable for determining antioxidant compounds, such as isoflavones, in the samples. The ethanolic extraction method employed was simple, rapid, and effective in extracting isoflavones, predominantly in the aglycone form. The concentration of isoflavones in the pulse-based ingredients used in the samples, namely isolated soy protein and white bean, aligned with the data in the literature for total isoflavone content in processed soy-based foods, such as protein isolates. Based on international guidelines, the detected isoflavone levels in the samples were deemed safe for daily consumption, considering the limits established to prevent allergies in children and hormone-related issues in adults. Another relevant aspect is that the voltammetric technique did not detect the natural dyes added to the food inks, either due to the non-electroactive nature of the molecules under the specified analysis conditions or their low solubility in 70% ethanol. The assessment of antioxidant activity through the calculation of the electrochemical index proved useful and straightforward for comparing samples subjected to the same conditions. However, for comparative purposes with data from the literature, this method may not be adequate due to the robustness of data obtained using other techniques. Nevertheless, despite the limited literature on the subject, a correlation between the electrochemical index and the EC50 has been observed. The voltammetric method utilizing a bare glassy carbon electrode proved effective in achieving the objectives of this study. Nonetheless, future investigations should focus on enhancing the efficiency of the presented voltammetric method by reducing the analysis time and streamlining the electrode cleaning process. To this end, the use of laboratory-made portable electrodes with modified working surfaces incorporating metallic nanoparticles holds promise. In conclusion, our study contributes to the advancement of quality control methods for plant-based foods, specifically fish and seafood analogs. By employing voltammetry with a bare glassy carbon electrode, we successfully quantified antioxidant compounds in the samples. This research opens avenues for the further exploration and optimization of electrochemical analysis techniques, paving the way for improved functional quality assessment in the rapidly growing field of plant-based meat analogs.

## Figures and Tables

**Table 1:** Validation parameters for determining genistein using differential pulse voltammetry and glassy carbon electrodes.

Parameters	Genistein
Curve equation	Y(µA) = 0.1959 × (µmol L^−1^) − 0.0163
Linearity range/Working range (µmol L^−1^)	0.5–3.0
Regression coefficient	0.9974 (*p* < 0.0001)
Cochran’s test	Ccalc (0.60) < Ctab (0.62) α = 95%; *N* = 6; *n* = 3
Detection limit (µmol L^−1^)	0.1
Quantitation limit (µmol L^−1^)	0.5
Repeatability (%) (*N* = 3; *n* = 7)	4.8 (0.5 µmol L^−1^) 5.4 (1.5 µmol L^−1^) 5.6 (3.0 µmol L^−1^)
Intermediate precision (%) (*N* = 3; *n* = 3)	7.4 (0.5 µmol L^−1^) 9.6 (1.5 µmol L^−1^) 8.8 (3.0 µmol L^−1^)

*N* number of concentration levels. *n* number of replicates per concentration.

**Table 2:** Concentration of total phenolics (mg GEN 100 g^−1^) and antioxidant activity (electrochemical index) in caviar and salmon analog samples.

Samples	mg GEN/100 g−1	EI (µA/mV)
Caviar analog	31.5 ± 1.8 (*n* = 5)	0.15 ± 0.01
Salmon analog	64.3 ± 2.0 (*n* = 5)	0.20 ± 0.01

## Data Availability

Data is unavailable due to intellectual property subjects.
